# Verification in Referral-Based Crowdsourcing

**DOI:** 10.1371/journal.pone.0045924

**Published:** 2012-10-10

**Authors:** Victor Naroditskiy, Iyad Rahwan, Manuel Cebrian, Nicholas R. Jennings

**Affiliations:** 1 Electronics and Computer Science, University of Southampton, Southampton, United Kingdom; 2 Masdar Institute of Science and Technology, Abu Dhabi, United Arab Emirates; 3 School of Informatics, University of Edinburgh, Edinburgh, United Kingdom; 4 Department of Computer Science and Engineering, University of California at San Diego, La Jolla, California, United States of America; 5 National Information and Communications Technology Australia, Melbourne, Victoria, Australia; 6 Department of Computing and Information Technology, King Abdulaziz University, Jeddah, Saudi Arabia; University of Namur, Belgium

## Abstract

Online social networks offer unprecedented potential for rallying a large number of people to accomplish a given task. Here we focus on information gathering tasks where rare information is sought through “referral-based crowdsourcing”: the information request is propagated recursively through invitations among members of a social network. Whereas previous work analyzed incentives for the referral process in a setting with only correct reports, misreporting is known to be both pervasive in crowdsourcing applications, and difficult/costly to filter out. A motivating example for our work is the DARPA Red Balloon Challenge where the level of misreporting was very high. In order to undertake a formal study of verification, we introduce a model where agents can exert costly effort to perform verification and false reports can be penalized. This is the first model of verification and it provides many directions for future research, which we point out. Our main theoretical result is the compensation scheme that minimizes the cost of retrieving the correct answer. Notably, this optimal compensation scheme coincides with the winning strategy of the Red Balloon Challenge.

## Introduction

Social networks facilitate efficient and fast search for rare information [1]–[6]. This is accomplished as individuals who are already involved in the search, share their quest with their friends, in effect referring them. We term this type of crowdsourcing *referral-based*. Providing every member with incentives to recruit as well as participate in the search opens enormous possibilities for rallying people for a particular cause [7].

To this end, a scientific study of the power of social networks and media to mobilize human populations was undertaken by the United States Defense Advanced Project Research Projects Agency (DARPA) in 2009. In the DARPA Network Challenge (also known as the Red Balloon Challenge) 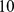 red weather balloons were placed at undisclosed locations throughout the United States. Participating teams competed to be the first to locate all of the balloons and win a prize of 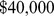. The lessons learnt from the DARPA Network Challenge, both from the scientific and practical standpoints, are almost solely drawn (with few exceptions, e.g. [8]) from the different team strategies to maximize the awareness and subsequent enrollment into the search by the different competing teams [9]. These strategies ranged from relying on people's altruism to help in the search, to web-based marketing to large communities of interest, to pure financial incentives [9].

However, recruiting people is only half of the story. The other half is distinguishing accurate balloon submissions from inaccurate ones. For instance, the majority of submissions of balloon sightings to the winning MIT team turned out to be false (either by sabotage or by mistake), and the verification task turned out to be the most challenging, time consuming, and likely the single most decisive factor in the competition [10]. In MIT's case, this task was performed by a time-consuming mixture of common-sense geo-location rules and direct verification by establishing direct communication with the participants [9]. Whereas the MIT recruitment mechanism has been described and analyzed in [7] and further studied in [11], little is known about the adequacy/optimality of its verification strategy, or any other team's approach.

In this paper, the crowdsourcing process is expanded to include *verification*: i.e., the ability to check the accuracy of reports and to filter out false ones. In other words, not only information gathering, but also verification is crowdsourced helping filter out false submissions before they reach the root. It is important to note that the problem of verification is in no way specific to the DARPA Network Challenge, but a subject of current research in crowdsourcing tasks including content annotation [12]–[14], user recommendations [15], and disaster relief [16].

In particular, this work initiates a formal study of verification in crowdsourcing settings where information is propagated through referrals. We propose a model which is simple and yet illustrates issues that we believe remain salient in many realistic information gathering scenarios such as maps of human-rights violations or post-disaster damage reports. In our model, each referred participant submits a false report with a given probability. Each report can be verified at a cost by the person who referred the reporting participant. Reports returned to the root may or may not have been confirmed to be accurate. Should a false report make its way to the root, the recruiter who failed to verify the report is penalized. Within this model, we derive the compensation scheme that minimizes the amount of reward necessary to recover the true answer. Notably, the optimal payment scheme is the same as the 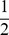-split contract used by MIT in the DARPA Network Challenge, though the team did not have the benefit of this analysis in setting their actual strategy.

The rest of the paper is structured as follows. We highlight the need for verification using the DARPA Network Challenge as an example. After that, we present a model incorporating false reports and the possibility of verification. Analysis of the minimum required reward and penalty follows together with a proof of the optimality of the 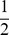-split/MIT contract. Finally, we review related work and provide conclusions and directions for future work.

### The DARPA Network Challenge

Our motivating example is the DARPA Network Challenge [7], [9]. This challenge required teams to provide coordinates of 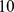 red weather balloons placed at different locations in the continental United States, offering a reward of 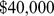 to the first team to report all correct locations.

This large-scale mobilization required the ability to spread information about the tasks widely and quickly, and to incentivize individuals to act. The MIT team completed the challenge in 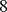 hours and 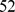 minutes. In approximately 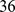 hours prior to the beginning of the challenge, the MIT team was able to recruit almost 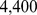 individuals through a recursive incentive mechanism.

**Figure 1:**
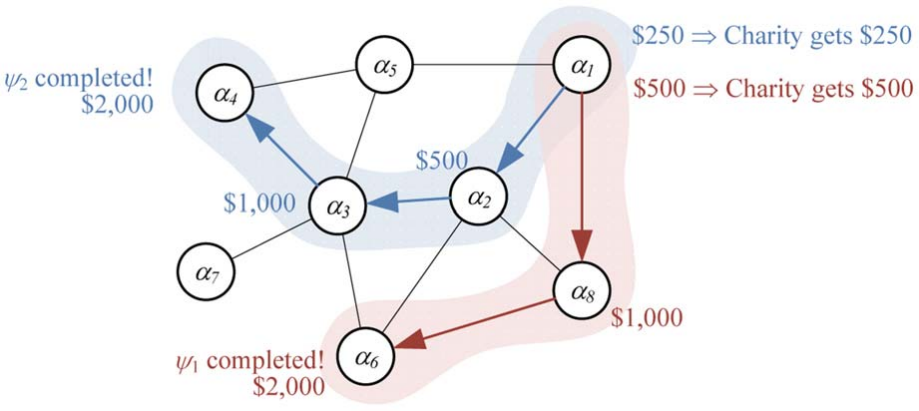
Recruitment tree with two paths (shown in thick lines) initiated by 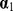 led to finding balloons.

The MIT team's approach was based on the idea that achieving large-scale mobilization towards a task requires diffusion of information about the tasks through social networks, as well as incentives for individuals to act, *both* towards the task and towards the recruitment of other individuals. This was achieved through a *recursive incentive mechanism*, which is illustrated in Figure 1. The mechanism distributes up to 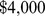 per balloon to people along the referral path that leads to the balloon. The person who finds the balloon gets 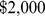, his immediate recruiter (or, *parent*) gets one half of the finder's compensation, etc. In Figure 1, agent 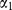 recruits all of his neighbors, namely 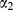, 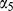 and 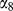, while agent 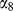 recruits 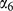, who finds balloon 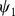. The finder receives 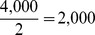. Since 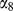 recruited 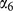, it gets 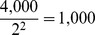. From this sequence, 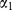 receives 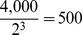.

Likewise, looking at the left recruitment path, the finder receives 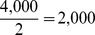. As above, we have 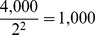 for 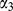 and 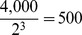 for 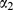. From this sequence, 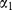 receives 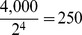. Adding up its payments from the two sequences it initiated, 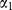 receives a total payment of 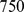. Notice that the amount distributed to the agents never exceeds 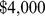 per balloon. In this example 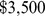 was paid for the first balloon and 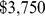 — for the second. The MIT team donated the undistributed money to charity.

The contracts described above can be dubbed *split contracts*, specifying the percentage of total child's reward that must be passed back to the parent. In particular, the MIT's winning strategy used a 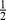-split contract across all nodes.

**Figure 2:**
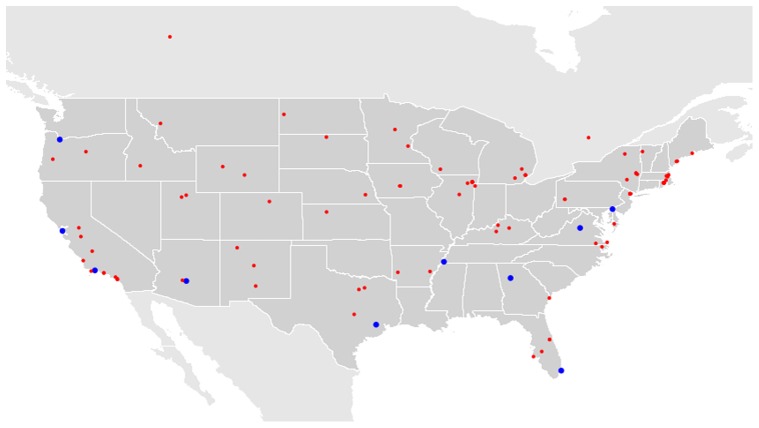
Reports of balloons sightings during the Red Balloon Challenge. Ten big circles represent the true reports. The small circles are for the false reports.

However, as mentioned above, the MIT's strategy assumed that any balloon citing report is correct. Yet, in the actual challenge, verifying balloon reports turned out to be a major obstacle [10]. Indeed, 124 out of 186 reports turned out to be false either by sabotage or by mistake. Figure 2 shows all of the reported locations, highlighting the prevalence of false reports in this kind of time-critical task (see [9] for examples of misreports). In this paper, we make a first attempt to model and tackle the verification problem.

## Results

### Modeling Referral-Based Crowdsourcing

We model scenarios where the *center* has an information gathering task. The information-seeking entity is represented by the root of a tree, and each node in the tree holds the answer required to complete the task with a fixed probability 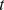. Information about the task (or the question) is propagated through referrals sent from parents to their children until a node holding the answer is found. The nodes along the path are compensated to ensure that once an answer-holding node is reached, it reports the answer (thus, 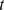 is the probability of holding *and returning* the answer). Unlike the other models, we allow for the possibility of false reports.

A crucial modeling choice regards the cause of the false reports. A rational agent that has no stake in the mechanism except for the compensation received, has no incentive to lie as false reports never result in a compensation. One may consider a richer population of agents where some agents derive utility from the mechanism not succeeding in recovering the true answer, or from increasing the time until a true answer is discovered. Designing a compelling utility function for this “lying” type of agent is an option. However, in such models, false reporting can be overcome by a payment that is high enough to make the agents' utility from truthful reporting a better option.

Given this, we pursue a different, simpler modeling avenue which does not rely on agent utilities. Instead, with probability 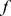 each node happens to be “irrational”. Such a node does not hold the true answer, but claims that he does and sends a false report to its parent. In other words, 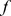 is the probability that the node does not hold the answer and generates a false report. These irrational agents are not affected by penalties and compensation: they lie irrespective of the incentives. In this model, misreports cannot be prevented but have to be discovered. Such a model of misreports is consistent with ignorant rather than malicious behavior: e.g., misreports due to mistakes and noise. As we alluded to earlier, modeling malicious behavior requires making assumption about the utility functions of the malicious agents, and it remains an avenue for future research. A good starting point may be the work on spiteful bidding (e.g., see [17], [18]).

As soon as a “reporting” node is recruited and generates an answer (correct or mistaken), it can no longer recruit other nodes as, from its point of view, the answer has already been recovered. Therefore, a reporting node is always a leaf node and only leaf nodes can generate false reports.

Now that we settled on how false reports arise, we need to model the verification process. We are going to assume that a node other than the reporter can verify the report with certainty. Naturally, verification requires some effort which we model with the cost 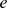 incurred by the node performing verification. Note that under the “perfect” verification, it is sufficient to obtain just one verification. The next question is which node should perform the verification. The most immediate candidate is the parent of the reporting node. After all, it is the parent node who decides which children to invite, and it is reasonable to hold him accountable for his invitees. Also, from the point of view of invited children, the first point of task-related contact for them is the parent. Furthermore, nobody except for the recruiter may have the authority/ability to question the recruit.

Given the assumptions above, we model the sequence of events next. A report goes from the reporting node to the node who recruited it — its immediate parent in the referral tree. On receiving a report, the immediate parent can verify whether the report is correct incurring the cost 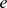. If the report is verified to be false, it is dropped. Otherwise, the immediate parent submits it directly to the root. To encourage verification, we assume the mechanism supports penalties. If a false report is propagated from a leaf node 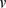 back to the root, the immediate parent of 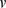 has to pay the penalty 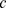. Penalizing the leaf node does not make sense as a node submitting a false report is irrational and, thus, indifferent to monetary incentives. Penalizing ancestors other than the immediate parent is not fair as they cannot verify the report or control its submission to the root.

Following [11], we propagate rewards using split contracts (we discuss why the choice of split contracts is justified in the Related Work section). Suppose node 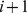 has the correct answer. Let 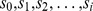 refer to split contracts offered on the path from the root node 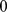 to node 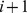: i.e., the root offers 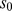-split to the first node on the path, who offers 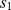-split to the second node, etc. The fraction of the reward received by each node is shown in Table 1.

**Table 1:** Distribution of the reward 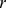 under a split contract.

node 1 receives	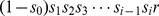
node 2 receives	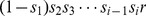
…	
node 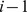 receives	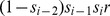
node 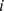 receives	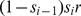
node 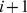 receives	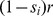

We will be concerned with incentivizing the immediate parent 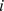 of the reporting node 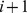 to participate and perform verification. His share of the reward is 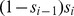. For example, under the 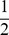-split contract, the parent of a reporting node receives a quarter of the reward.

### Optimal Mechanism

The model detailed above is specified by the probabilities of false and true reports, the verification cost, the penalty level, the reward provided by the root, and the split contract determining allocation of the reward (see Table 2). While the first 3 parameters are exogenous, the root is likely to have control over the penalty level, as well as distribution of the reward. Clearly, it is in the root's interest to minimize the reward given out. In this section we derive the split contract which minimizes the reward required to recover the answer. We also describe the penalty level sufficient to ensure that verification takes place and no false reports are propagated.

**Table 2:** Symbol list.

Symbol	Meaning
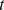	Probability that a node has the answer
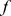	Probability of a node generating a false report
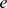	Verification cost
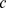	Penalty for submitting a false report
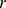	Reward offered by the root node
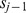	Percentage of its reward that node 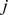 must pass to parent 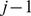

Recall that 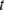 and 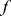 refer to the probabilities of submitting true and false answers respectively. These events are disjoint and the probability that a report is correct is 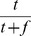. Let 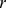 denote the reward offered by the root. Consider a reporting node 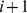 and his parent 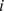. If the report is correct, the parent will receive 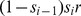 resulting in the expected reward of 
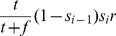
. When deciding on whether or not to verify the report, the parent must consider the verification cost 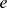, and the penalty 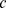 for propagating false reports. Verification cost is incurred regardless of the report accuracy, while the penalty is paid only if the report is false. The utility of the parent performing verification appears on the left hand side while the utility when no verification is performed is on the right:
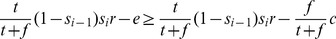
(1)Thus, the parent prefers verifying the report when the verification cost is below the expected penalty 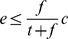.

#### Proposition 1

*For a fixed* 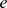, *the minimum level of penalty that enforces verification is*

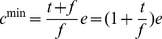
(2)We say that the *verification constraint* is satisfied if the penalty is at least the minimum penalty. Notice that whenever the constraint is satisfied, verification will take place and no penalty will be incurred.

Not surprisingly, the minimum penalty is proportional to verification costs. Somewhat paradoxically, however, the required penalty is highest when almost all reports are true: i.e., when the ratio 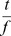 is high. The reason for this is that from the point of view of the parent, verification is going to be a wasted effort as there is a high probability the report is true. Viewed differently, the chances of being penalized for propagating an unverified report are small. Thus, the penalty must be high enough to counteract these effects and eliminate all incorrect submissions.

Verification is most important when the number of false reports is large relative to the number of true reports. For example, during the Red Balloon Challenge, the MIT team received 186 reports with only 62 being true (see Figure 2). A high number of false reports is likely in scenarios where the answer is difficult to locate requiring a large number of nodes to be explored. Note that these are exactly the scenarios relevant to referral-based crowdsourcing. The expected number of reports until a true report is submitted is given by the mean 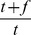 of the geometric random variable specified by the success probability of 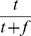. For example, when 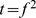, the expected number of false reports is 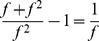.

The minimum penalty level provides incentives for the agents to verify the report rather than propagate it directly. However the agents have another option, which is to not participate at all. In order to encourage participation, the reward has to be high enough as we discuss in the next paragraph. First, we observe that a parent node also has an option to ignore a report that needs verification in the hope that the answer will be found and verified by other nodes deeper down his subtree. We assume this strategy never leads to a positive payoff (for example, this is the case if the reporting node complains that his report is held due to the parent's reluctance to verify, resulting in the parent being disqualified).

To encourage participation (assuming the verification constraint (1) is satisfied), the parent's expected utility must be non-negative 
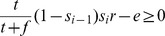
; i.e., the expected reward must exceed the effort. Rearranging the terms of this inequality, we get the following participation constraint for node 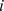.

#### Proposition 2

*The minimum reward sufficient to encourage participation of node* 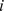 *is*

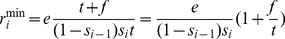
(3)Not surprisingly, higher verification costs require higher rewards. In contrast to the minimum penalty, the required reward increases with the ratio of false reports to true reports 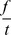 (i.e., decreases with 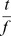). Intuitively, the required reward is proportional to the cost of verification incurred before a true answer is found. When the probability of false reports is high relative to the probability of true reports, the total verification effort spent before a true answer is discovered is high. The proposition above assumes the root never receives more than one true report at the same time, and once the true report is received, all nodes are immediately made aware of it and do not incur any costs by performing verification after that. Multiple true reports can be allowed without affecting the incentives by compensating nodes along each path independently. Of course, this means spending the required reward multiple times, once for each true report.

The reward required to satisfy the participation constraint (3) for the parent of a reporting node 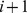 is inversely proportional to the fraction 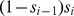 of the reward that the parent receives. If we knew *a priori* which nodes would be reporting answers, we could minimize the required reward by giving all of it to the immediate parents (immediate parents are the only nodes with non-trivial verification and participation constraints as only they can perform verification and incur penalties). However, any node could be the immediate parent, and contracts must be designed without knowing which nodes will initiate a report. In other words, the participation constraint must be satisfied for any node
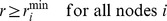
(4)Notice that unlike the results for the other referral models [11], [19], the reward does not depend on the depth to which the tree needs to be explored. This is due to the lack of cost for propagating the answer – which may be a more realistic assumption.

Next we find the contract that minimizes the required reward 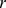.

#### The Optimal Split Contract

The MIT mechanisms (i.e., 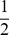-split) is a special case of the family of split-contract mechanisms. While intuitively the 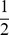-split seems to be the most natural one, no theoretical guarantees on its performance have previously been provided. We do this here. As we will show, in the context of our model, the 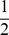-split mechanism is the optimal split-contract mechanism.

#### Theorem 1

*The* 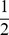 *-split contract minimizes the reward required to recover the answer.*

#### Proof

Suppose node 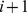 returns the true answer and recall the corresponding distribution of the reward in Table 1. We are free to choose the values for 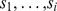 that minimize the required reward subject to the participation constraint. Specifically, at the time the contract is offered to node 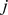 on the path to node 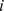, it must hold that 
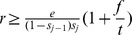
. The required reward is inversely proportional to 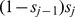. Also, observe that the constraint must hold for any 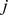, and thus, the required reward is determined by the node 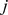 with the lowest share 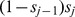. Formally, the reward required by a contract 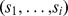 is given by the minimum value 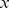 that satisfies
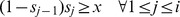
In fact, a split-contract must specify shares 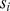 for any 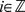, since the mechanism must return the true answer no matter how deep in the tree it is found.
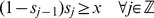
(5)It is easy to see that 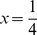 for the 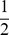-split mechanism (i.e., 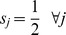). Next we show that no other mechanism can have a higher performance guarantee. Suppose 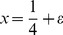 for 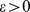. Constraints (5) can be written as
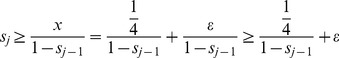
We used 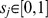 to obtain the last inequality. Observe that 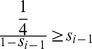 for 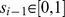 to obtain
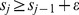
Since the above inequality holds for all 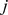, we get
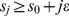
But for any 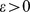 this results in 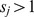 for 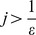 violating the constraint 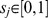. Thus, 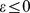 and 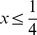, establishing optimality of the 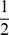-split. 

## Discussion

### Related Work

Our work can be seen as an application of mechanism design [20] to social networks and information gathering tasks. The model has similarities to the model of Query Incentive Networks (QIN) presented by Kleinberg and Raghavan [19]. In that model, the root needs to recover an answer from a network of nodes where each node has a small probability of holding the answer. In order to encourage nodes to return the answer, the root proposes a reward that is propagated down the tree. Once an answer-holding node is recruited, it sends the answer to its parent, who forwards it to the grandparent, and so on until the root is reached. There is a constant (integer-valued) cost incurred by each node on the path from the answer-holding node back to the root. The authors describe the minimum reward required to obtain the answer with high probability when each parent can offer a reward to its children.

Our model is similar to the QIN model in that we are searching to retrieve an answer from the network where the question is propagated via invitations that parents send to their children. The main novel ingredients in our model are (i) the possibility of false reports; (ii) the option to verify the reports at a cost; and (iii) the ability of the root to penalize false report submissions. These attributes appear in many real-world settings making our model more readily applicable.

It is interesting to note that the introduction of costly verification and penalties allowed us to dispense with one of the assumptions of the QIN model: the costly propagation of the answer is no longer required. Without this assumption, the QIN model admits degenerate solutions, where the root gets the answer for an arbitrarily small cost (indeed, the required reward would also be zero in our model if we set the probability of false reports to zero). Our disposal of this assumption is important, for example in situations where propagating the answer has negligible cost (e.g., forwarding an email or re-tweeting) relative to a demanding verification task (e.g., checking if a balloon report is authentic by personally sighting it), or when the nodes can send the answer directly to the root without propagating it up the referral path.

Our model restricts attention to split contracts. However, this seems to be the right class of contracts to focus on for the following reasons. The simplest and most common alternative is fixed rewards: each parent promises its children a fixed amount. While original work on QIN considered fixed contracts [19], Cebrian *et al.* [11] showed that a significantly lower reward is required when using split contracts. Also, in our setting, where verification and recovery of the answer must occur with certainty, fixed contracts are inappropriate: any contract that offers a fixed amount to each node will require an infinite investment to be recovered with probability one as the answer may be arbitrarily deep within the tree. Another natural idea is to share the reward equally among all the nodes along the path. In this context, this division of the reward coincides with the Shapley value [21]. However, such a division would also require an infinite reward in our model: for an answer that is 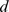 levels deep, the root would have to pay *each* of the 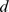 nodes along the path the minimum reward that a node will expect to undertake verification. Notice that 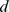 may be arbitrarily large.

Another justification of split contracts comes from the work of Emek *et al.* [22]. The authors take an axiomatic approach to show that a special case of split contracts with an equal split at each level arises naturally in multi-level marketing. Similar to our model, in multi-level marketing recursive referrals are sought from the participants. A fundamental difference is that in the marketing model participants are compensated for each referral they make, while in QIN and our context, only referrals that contribute to finding the answer generate a reward.

We acknowledge that in reality recruitment trees are finite and potentially not very deep, while our assumption is that propagation of referrals can produce arbitrarily deep trees, reaching very large (possibly infinite) numbers of individuals. Indeed, recent work suggests that we live in a “small but slow world”, as social network topology and human burstiness can actually hinder information propagation, effectively reducing the population reached [23]–[28]. However, as the DARPA Network Challenge showed, wide dissemination of information does occur in certain scenarios [29]–[33].

### Conclusions and Future Work

Since the seminal experiments by social psychologist Stanley Milgram in the 1960s, it has been established that social networks are very effective at finding target individuals through short paths [1]. Various explanations of this phenomenon have been given [3], [4], [6], [34]. However, it has also been recognized that the success of social search requires individuals to be motivated to actually conduct the search and participate in the information diffusion. Indeed, it has been shown experimentally that while successful chains happen to be short, the majority of chains observed empirically terminate prematurely [5]. Dodds *et al.* conclude that “*although global social networks are, in principle, searchable, actual success depends sensitively on individual incentives*” [5]. In other words, a key challenge in social search is the *incentive challenge*. However, while models like the Query Incentive Networks model [19], and the split-contract approach [7] both provide incentives for diffusion, the problem of verifying the received reports has not previously received any formal treatment.

The issue of verification arises in many real-world crowdsourcing scenarios (e.g., mapping social uprisings [35], [36] or gathering disaster response requests [37], [38]). Indeed, some competitive scenarios have even been subject to larger levels of sabotage, as illustrated by the attack on the crowdsourced strategy to tackle the 2011 DARPA Shredder Challenge [39]. For such settings, our paper provides the first steps towards formally analyzing verification schemes. Specifically, we introduced a model for studying verification in referral-based crowdsourcing. We explored the relationship between various parameters, including the size of the reward offered by the mechanism, the probability of possessing the answer, the probability of false reports, the cost of verifying the correctness of reports, and the penalty imposed by the mechanism on false reports. Our main theoretical result is the proof that the optimal distribution of the reward in our model is given by the 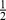-split contract. This contract happened to be the one used by the winner of the Red Balloon Challenge, showing that this way of sharing the reward is also appealing in practice. Our paper provides the first theoretical justification of this mechanism in the presence of misinformation. Our second contribution is in initiating a formal study of verification in information-gathering scenarios. Our model provides a starting point for future research where various assumptions may be relaxed. We outline some directions next.

We provided results for the uniform and known verification cost. Bitcoin provides an example of a real system where this assumption holds: the expected computational cost of authorizing a transaction is uniform and known (see [40] for more details on Bitcoin). In other scenarios such as quality verification of crowdsourced tasks (e.g., accuracy of a translation, deciding whether a photo is authentic, or evaluating a programming job) costs may be heterogeneous as well as the private information of the agents. Our model can be directly extended to introduce heterogeneous and private costs for the analysis of such scenarios.

In online scenarios one may easily create multiple identities. Thus, it is particularly important to consider mechanisms that are resilient to coalitions of lying nodes. Since coalitions may easily control an entire referral path, verification from nodes outside the path is likely to be required. This question has been tackled with “uniform” rather than split contracts in [40]. Moreover, for split-contracts, some lessons may be drawn from the results on false-name-proof mechanisms for multi-level marketing [22].

It is also interesting to weaken some of our other modeling assumptions. For example, if we no longer assume that the probability that a node holds the answer (or lies) is uniform, the mechanism should encourage the recruitment of individuals more likely to possess the answer, perhaps based on the knowledge that agents have about the abilities and reliability of their peers. An even more selective recruitment is likely to arise if the cost of recruiting others is non-zero.

Our model makes the assumption that the split contract (e.g., 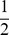-split) is selected by the mechanism and cannot be modified by other nodes. This contrasts with the models of [11], [19], where each node chooses which contract to offer to its children, and the resulting equilibrium contract is analyzed. An important direction for further study, therefore, is to perform equilibrium analysis in our model, when nodes not only choose who to recruit and whether to verify, but also what split to offer.

Finally, an important extension of our work is to explore the dynamics of strategic behavior in the context of repeated interaction. In particular, a threat of non-monetary punishment may be sufficient to encourage verification. For example, in permanent systems such as Amazon Mechanical Turk, Wikipedia or Bitcoin, the penalty may be imposed in the form of decreased reputation, which diminishes future earning potential or the influence a user exercises [41].
